# Survival of people with valvular heart disease in a large, English community-based cohort study

**DOI:** 10.1136/heartjnl-2020-318823

**Published:** 2021-05-24

**Authors:** Clare J Taylor, José M Ordóñez-Mena, Nicholas R Jones, Andrea K Roalfe, Saul G Myerson, Bernard D Prendergast, FD Richard Hobbs

**Affiliations:** 1 Nuffield Department of Primary Care Health Sciences, University of Oxford, Oxford, UK; 2 Division of Cardiovascular Medicine, Radcliffe Department of Medicine, University of Oxford, Oxford, UK; 3 Department of Cardiology, St Thomas’ Hospital, London, UK

**Keywords:** heart valve diseases, atherosclerosis, echocardiography, epidemiology

## Abstract

**Objective:**

Valvular heart disease (VHD) is present in half the population aged >65 years but is usually mild and of uncertain importance. We investigated the association between VHD and its phenotypes with all-cause and cause-specific mortality.

**Methods:**

The OxVALVE (Oxford Valvular Heart Disease) population cohort study screened 4009 participants aged >65 years to establish the presence and severity of VHD. We linked data to a national mortality registry and undertook detailed outcome analysis.

**Results:**

Mortality data were available for 3511 participants, of whom 361 (10.3%) died (median 6.49 years follow-up). Most had some form of valve abnormality (n=2645, 70.2%). In adjusted analyses, neither mild VHD (prevalence 44.9%) nor clinically significant VHD (moderate or severe stenosis or regurgitation; 5.2%) was associated with increased all-cause mortality (HR 1.20, 95% CI 0.96 to 1.51 and HR 1.47, 95% CI 0.94 to 2.31, respectively). Conversely, advanced aortic sclerosis (prevalence 2.25%) and advanced mitral annular calcification (MAC, 1.31%) were associated with an increased risk of death (HR 2.05, 95% CI 1.28 to 3.30 and HR 2.51, 95% CI 1.41 to 4.49, respectively). Mortality was highest for people with both clinically significant VHD and advanced aortic sclerosis or MAC (HR 4.38, 95% CI 1.99 to 9.67).

**Conclusions:**

Advanced aortic sclerosis or MAC is associated with a worse outcome, particularly for patients with significant VHD, but also in the absence of other VHD. Older patients with mild VHD can be reassured about their prognosis. The absence of an association between significant VHD and mortality may reflect its relatively low prevalence in our cohort.

## Introduction

Valvular heart disease (VHD) is a common condition affecting up to one in two adults aged over 65 years.^1^ Prevalence is predicted to increase due to population ageing and the growing prevalence of risk factors.^2^ Moderate or severe VHD impacts on cardiac function, is associated with impaired quality of life and increased hospitalisation,^3^ and can progress to heart failure, arrhythmias and death.^4^

There is no effective medical therapy,^5^ but timely identification of individuals with severe VHD who may benefit from valve intervention can reduce or eliminate symptoms, prevent complications and improve outcome.^6^ In contrast, mild VHD does not require intervention and its importance is uncertain. Previous secondary care studies suggesting an impact of mild aortic valve disease on outcome may not be applicable to larger community populations.^7^ The true impact of VHD on life expectancy and whether death is due to heart-related problems or other conditions are therefore unknown.

The OxVALVE (Oxford Valvular Heart Disease) study, a large community-based echocardiographic screening programme, was established in 2009.^8^ Newly identified VHD was found in 1269 of the first 2500 participants (50.8%).^9^ Most were mild, with only 159 participants (6.4%) having a new diagnosis of moderate or severe disease on screening and a further 4.9% of patients at participating practices having pre-existing moderate or severe VHD. The total prevalence of moderate or severe VHD in the population was therefore 11.3%.

The OxVALVE-Survive study aimed to report the survival rates of people in the OxVALVE cohort with and without VHD, determine their cause of death, and investigate the association of VHD and its various phenotypes with all-cause and cause-specific mortality.

## Methods

### Study design and population

The OxVALVE database was used to identify people with and without VHD. In total, OxVALVE recruited 4009 participants from seven general practices in England between August 2009 and May 2016. All people registered with each practice aged over 65 years without a diagnosis of VHD were eligible to participate and invited for echocardiography. Transthoracic echocardiography was carried out prospectively by a British Society of Echocardiography accredited echocardiographer.

All participants provided written informed consent and 3515 gave permission for their records to be linked to the Office for National Statistics (ONS) civil death registry, using participants’ NHS Digital number and date of birth.

### Predictor and outcome definitions

VHD was categorised using international criteria as previously described,^9^ according to the affected valve, severity of valve lesion and associated clinical relevance (none/trivial, mild or clinically significant—the latter defined as moderate or severe stenosis or regurgitation). Primary and secondary mitral and tricuspid regurgitation were subcategorised using established nomenclature.^10^ We also examined the association between VHD and mortality in subjects with aortic sclerosis or mitral annular calcification (MAC)—both common valve abnormalities that have no functional effect but are markers of atherosclerosis and vascular disease.^11^ Aortic sclerosis and MAC were collectively defined as manifestations of calcific valve disease without functional effect. People with aortic sclerosis could not have coexistent aortic stenosis, but could have other VHD. People with MAC could have associated mitral stenosis or regurgitation, or other VHD. Classification was made according to a modified version of the American College of Cardiology and Nishimura/Otto criteria (online supplemental table 1).^12^

10.1136/heartjnl-2020-318823.supp1 Supplementary data

Information concerning vital status and cause of death was ascertained until June 2019. Cause-specific mortality was ascertained using International Classification of Diseases 10th revision codes as logged on the NHS Digital database. Causes of death occurring in less than 10 individuals were grouped.

### Statistical analyses

Characteristics of participants were described using summary statistics, according to VHD severity. Frequencies of single and combined VHD phenotypes were plotted using the R package ‘UpSetR’.^13^ Combinations occurring in more than 10 participants are shown. We estimated the associations with mortality for VHD phenotypes occurring in at least 100 participants.

Kaplan-Meier (KM) curves were used to display cumulative mortality and log-rank tests to compare groups. Restricted mean survival times were used to calculate the difference in expected survival time between groups.

Cox proportional hazards models were used to estimate HRs and their 95% CI for the association of VHD with all-cause mortality. We reported unadjusted and adjusted HRs for hypothesised confounder variables: age, sex, smoking status, socioeconomic status, rheumatic fever and atrial fibrillation. Covariates were only considered confounders, and therefore adjusted for if they were a cause of both exposure and disease. We did not adjust for variables that were considered either intermediate in the pathway between VHD and mortality (ie, a consequence but not cause of VHD) or prognostic variables (ie, related to mortality but not VHD) to prevent overadjustment bias.^14^ A simplified directed acyclic graph was used to summarise the variables considered (online supplemental figure 1).^15^ In sensitivity analyses, intermediate and prognostic variables were also adjusted for, but the estimates remained similar.

Two Cox regression models were used for the association of VHD phenotypes with all-cause mortality. Model 1 included all VHD phenotypes as predictors that were entered as two dummy variables (mild or clinically significant, with none/trivial as reference). Model 2 further adjusted for the hypothesised confounder variables. The proportional hazards assumption was tested by adding interaction terms to the model with subsequent graphical examination of Schoenfeld residual plots.

The Fine-Gray competing risks model was used to assess the relationship between VHD and cause-specific mortality using the same set of hypothesised confounder variables, with other causes of death modelled as a single competing outcome. Complete case analysis was used since the percentage of missing data was minimal (n=17, 0.48%).

All analyses were conducted with R V.3.6.0 (Vienna, Austria) using ‘survival’,^16^ ‘survminer’^17^ and ‘cmprsk’ packages.^18^

### Patient and public involvement

Lay participants contributed to the design and implementation of the OxVALVE study but were not directly involved in this analysis.

## Results

### Baseline characteristics and treatment

Linkage was possible for 3515 participants (median 6.49 years follow-up, maximum 9.73 years). Date of death was missing for one participant and three had missing echocardiographic information, leaving 3511 participants for analysis. The cohort in whom linkage was not possible (n=498) were more likely to be older (75.1 years vs 72.6 years) and male (56% vs 50%) with a history of cardiovascular disease (cerebrovascular event 9.0% vs 5.7%; hypertension 49.8% vs 44.3%) and a higher prevalence of mitral stenosis (2.2% vs 0.1%) and aortic regurgitation (18.7% vs 16.5%), but a lower prevalence of aortic sclerosis (40.4% vs 47.1%) and mitral regurgitation (23.5% vs 28.7%) (online supplemental table 2).

Among the included participants, any form of valve abnormality was present in 2645 (70.2%). VHD was present in 1760 (50.1%), of whom 1578 had mild VHD (44.9%) and 182 had clinically significant (moderate or severe) VHD (5.2%) (table 1; see online supplemental tables 3 and 4 for classification of VHD severity according to phenotype). Of the participants, 1878 (53.5%) had calcific valve disease without functional effect (either aortic sclerosis, MAC or both), of whom 833 (23.7%) had aortic sclerosis or MAC without other VHD (including 43 (1.2%) with advanced aortic sclerosis or MAC). In total, 288 (8.2%) had some form of advanced valve abnormality, either clinically significant VHD, advanced MAC or advanced aortic sclerosis.

**Table 1:** Baseline characteristics of the OxVALVE cohort according to the presence of valvular heart disease*

Characteristics	Valvular heart disease		
	No	Mild	Significant
Total	1751	1578	182
Age (years), mean (SD)	71.5 (5.1)	73.4 (6.3)	76.4 (7.2)
Sex, n (%)			
Male	829 (47.3)	850 (53.9)	90 (49.4)
Female	922 (52.7)	728 (46.1)	92 (50.6)
Medical history			
Myocardial infarction, n (%)	68 (3.9)	75 (4.8)	14 (7.7)
NYHA class, n (%)			
I	1422 (81.2)	1272 (80.6)	117 (64.3)
II	287 (16.4)	282 (17.9)	60 (33.0)
III	42 (2.4)	24 (1.52)	5 (2.8)
Angina, n (%)	126 (7.2)	120 (7.6)	15 (8.2)
Angiography, n (%)	116 (6.6)	136 (8.6)	30 (16.5)
Ankle oedema, n (%)	257 (14.7)	209 (13.2)	27 (14.8)
Atrial fibrillation, n (%)	70 (4.0)	114 (7.2)	40 (22.0)
Coronary artery bypass graft, n (%)	21 (1.2)	30 (1.9)	6 (3.3)
Cerebrovascular attack/transient ischaemic attack, n (%)	89 (5.08)	95 (6.0)	15 (8.2)
Diabetes, n (%)	232 (13.3)	141 (8.9)	18 (10.0)
Hyperlipidaemia, n (%)	686 (39.2)	552 (35.0)	67 (36.8)
Hypertension, n (%)	815 (46.5)	653 (41.4)	88 (48.4)
Percutaneous coronary intervention, n (%)	61 (3.5)	62 (3.9)	12 (6.6)
Rheumatic fever, n (%)	31 (1.8)	33 (2.1)	9 (5.0)
Smoking status, n (%)			
Non-smoker	848 (48.4)	925 (58.6)	110 (60.4)
Ex-smoker	768 (43.9)	567 (35.9)	61 (33.5)
Smoker	134 (7.7)	85 (5.4)	11 (6.0)
Index of multiple deprivation (quintile), n (%)			
1 (least deprived)	531 (30.3)	454 (28.8)	44 (24.2)
2	654 (37.4)	522 (33.1)	52 (28.6)
3	334 (19.1)	363 (23.0)	52 (28.6)
4	179 (10.2)	159 (10.1)	26 (14.3)
5 (most deprived)	44 (2.5)	75 (4.8)	8 (4.4)
Examination			
Height (m), mean (SD)	1.68 (0.10)	1.67 (0.09)	1.66 (0.10)
Weight (kg), mean (SD)	81.0 (16.2)	74.9 (14.6)	72.2 (14.6)
Body mass index (kg/m^2^), mean (SD)	28.6 (5.0)	26.9 (4.5)	26.0 (4.2)
Systolic blood pressure (mm Hg), mean (SD)	143.2 (19.5)	143.9 (20.6)	146.9 (23.0)
Diastolic blood pressure (mm Hg), mean (SD)	81.1 (11.2)	79.7 (11.3)	78.7 (13.3)
Heart rate (bpm), mean (SD)	74.6 (12.3)	71.1 (11.7)	70.8 (12.8)
Aortic sclerosis, n (%)			
None/trivial	1124 (64.2)	684 (43.4)	50 (27.5)
Early	600 (34.3)	850 (53.9)	124 (68.1)
Advanced (moderate/severe)	27 (1.5)	44 (2.8)	8 (4.4)
Mitral annular calcification, n (%)			
None/trivial	1545 (88.2)	1357 (86.0)	146 (80.2)
Early	190 (10.9)	199 (12.6)	28 (15.4)
Advanced (moderate/severe)	16 (0.9)	22 (1.4)	8 (4.4)
Valve intervention, n (%)			
Total	0 (0.0)	0 (0.0)	12 (6.6)
TAVI	0 (0.0)	0 (0.0)	5 (2.7)
Aortic valve replacement	0 (0.0)	0 (0.0)	4 (2.2)
Other	0 (0.0)	0 (0.0)	3 (1.7)

*Valvular heart disease includes mitral regurgitation, mitral stenosis, aortic regurgitation, aortic stenosis, tricuspid regurgitation, pulmonary regurgitation, bicuspid aortic valve and mitral valve prolapse.

bpm, beats per minute; NYHA, New York Heart Association; OxVALVE, Oxford Valvular Heart Disease population cohort study; TAVI, transcatheter aortic valve implantation.

Only 12 participants underwent valve intervention during follow-up, 7 of whom remain alive. The most common intervention was transcatheter aortic valve implantation (n=5).

### VHD phenotypes

**Figure 1:**
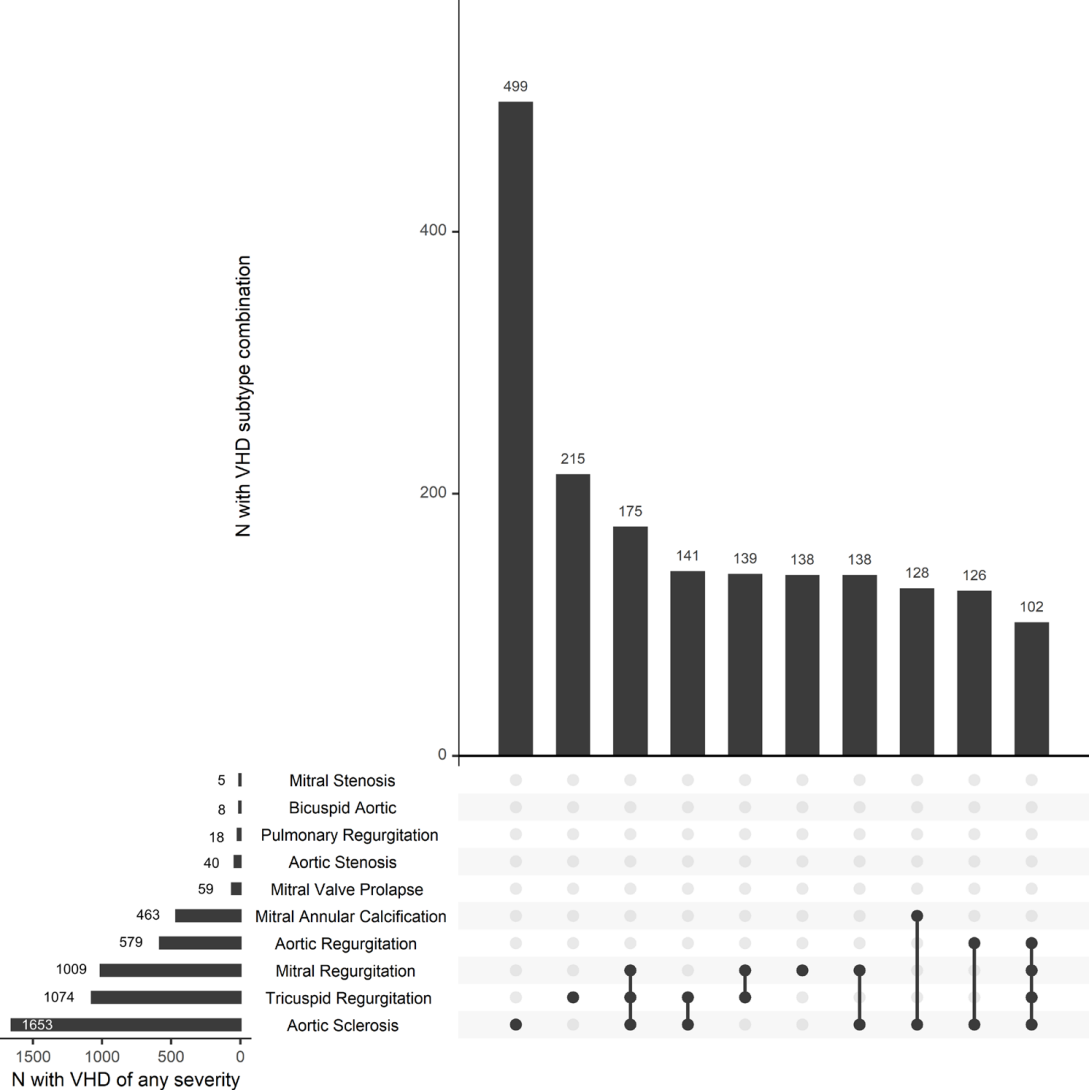
Frequencies of individual valvular heart disease (VHD) phenotypes, calcific valve disease without functional effect (aortic sclerosis or mitral annular calcification) and their combinations. The figure is a combination of three plots. The first (lower left panel) is a horizontal bar plot showing the frequency of all VHD subtypes considered. For example, aortic sclerosis is the most common phenotype and was present in more than 1500 study participants. The second plot (upper right panel) is a vertical bar plot showing the frequencies of the 10 most common combinations of VHD phenotypes. The third plot (lower right panel) shows the combinations of VHD phenotypes in the rows shown by the black dots. The lines linking the points denote the presence of more than one phenotype. A single dot indicates that the VHD phenotype occurred in isolation.

Tricuspid regurgitation was the most common VHD (mild: n=1009, 28.7%; moderate/severe: n=65, 1.85%) and aortic sclerosis the most common abnormality (early: n=1574, 44.8%; advanced: n=79, 2.25%) (figure 1). Multiple findings in individual patients were common, with aortic sclerosis and tricuspid regurgitation frequently occurring together or in combination with other VHD phenotypes (figure 1). Moderate/severe mitral regurgitation was predominantly of primary origin (primary 82%, secondary 18%). In contrast, moderate/severe tricuspid regurgitation was predominantly of secondary origin (primary 3%, secondary 83%, mixed primary/secondary 14%). Aortic sclerosis and MAC were more common among people with clinically significant VHD (table 1).

### Causes of death

By June 2019, 361 (10.3%) participants had died. Cancer was the most common cause of death (n=158), followed by cardiovascular (n=86) and respiratory (n=38) disease. Cardiovascular disease was listed as a contributing factor in almost half of all deaths (n=171, 47.4%) and more frequent in people with VHD (n=106, 51.5%) compared with those without (n=65, 42.8%) (online supplemental table 5), although these differences were not statistically significant in adjusted analyses.

### All-cause and cause-specific mortality in overall VHD cohort

Both prevalence and severity of VHD increased with age. Individuals with mild and clinically significant VHD were 2 and 5 years older on average, respectively, than those with no VHD.

**Figure 2:**
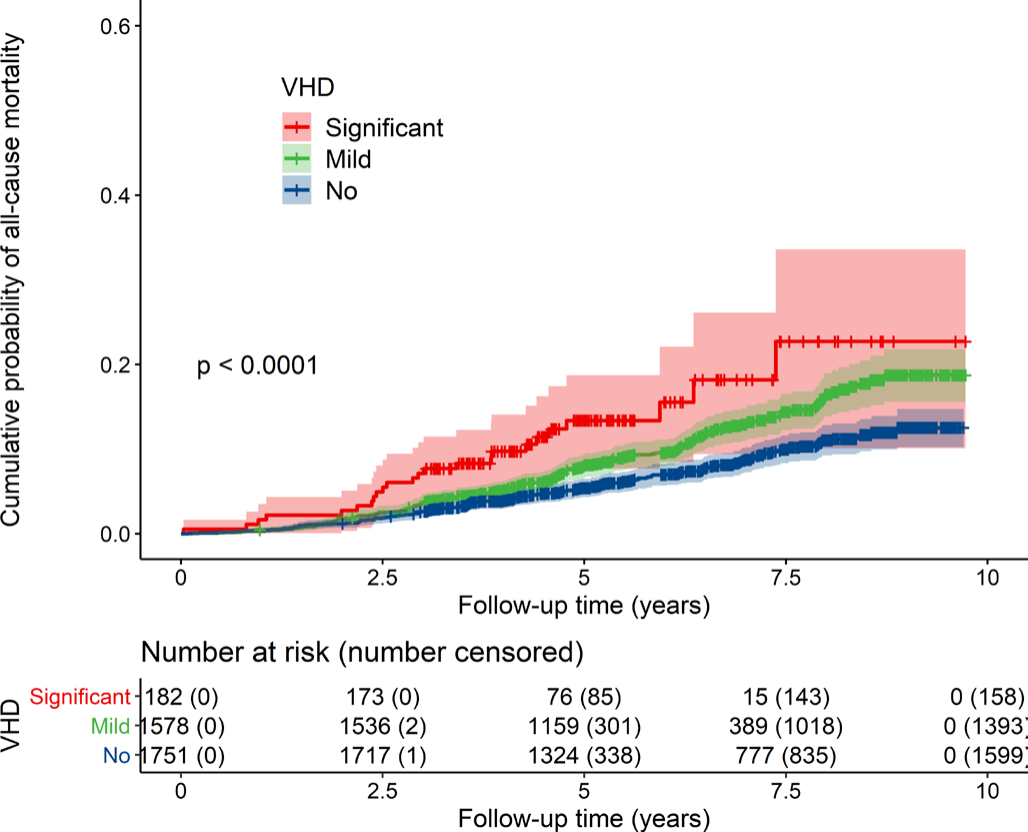
Cumulative probability of all-cause mortality in OxVALVE participants according to severity of valvular heart disease. The shaded area around each group line indicates 95% CI for the cumulative probability of all-cause mortality (p value=log-rank test). OxVALVE, Oxford Valvular Heart Disease; VHD, valvular heart disease.

In our unadjusted analyses, and excluding people with aortic sclerosis or MAC only, the risk of all-cause and cardiovascular mortality was increased in people with both mild and clinically significant VHD compared with those with no VHD (table 2). In KM analyses, people with either mild or clinically significant VHD had a reduced mean survival time compared with those without VHD (mild vs no VHD 3.0 months (95% CI 1.3 to 4.7, p<0.0001); clinically significant vs no VHD 7.6 months (95% CI 1.9 to 13.2, p<0.0001)) (figure 2). However, these differences were attenuated after adjustment for age and other variables (mild VHD HR 1.20, 95% CI 0.96 to 1.51; significant VHD HR 1.47, 95% CI 0.94 to 2.31, when compared with no VHD) (table 2).

**Figure 3:**
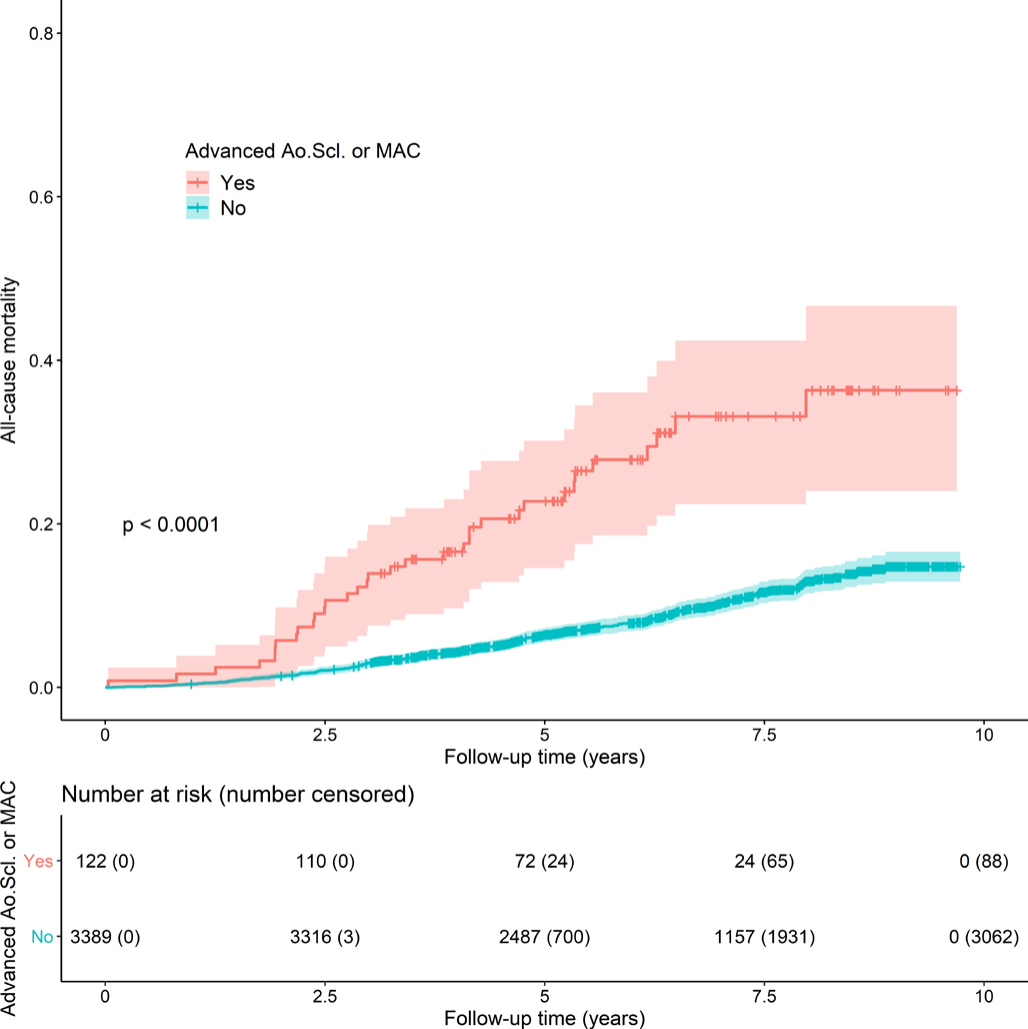
Kaplan-Meier curve demonstrating the unadjusted survival rates for people with advanced aortic sclerosis (Ao.Scl) or mitral annular calcification (MAC) compared with people with early or no disease. Participants are categorised as having advanced aortic sclerosis or mitral annular calcification (types of calcific valve disease without functional effect), irrespective of the presence of valvular heart disease. Advanced disease describes moderate or significant sclerosis or calcification, although without functional impact.

**Table 2:** Association of VHD stratified according to severity with all-cause and cause-specific mortality

Outcome VHD severity	N	n	PYs	MR	Crude model	P value	Multivariable model*	P value
All-cause mortality								
No	1751	152	11 508	13.2	1.00 (reference)		1.00 (reference)	
Mild	1578	185	9685	19.1	1.51 (1.22–1.87)	<0.001	1.20 (0.96–1.51)	0.10
Significant	182	24	878	27.3	2.43 (1.58–3.75)	<0.001	1.47 (0.94–2.31)	0.09
CVD†								
No	1751	34	11 508	3.0	1.00 (reference)		1.00 (reference)	
Mild	1578	44	9685	4.5	1.59 (1.02–2.49)	0.04	1.15 (0.72–1.83)	0.57
Significant	182	8	878	9.1	3.34 (1.54–7.26)	0.002	1.52 (0.63–3.68)	0.35
Cancer†								
No	1751	79	11 508	6.9	1.00 (reference)		1.00 (reference)	
Mild	1578	76	9685	7.8	1.14 (0.83–1.57)	0.40	1.05 (0.76–1.45)	0.77
Significant	182	3	878	3.4	0.50 (0.16–1.60)	0.24	0.37 (0.11–1.24)	0.11
Respiratory disease**†**								
No	1751	14	11 508	1.2	1.00 (reference)		1.00 (reference)	
Mild	1578	19	9685	2.0	1.60 (0.80–3.17)	0.18	1.12 (0.54–2.32)	0.76
Significant	182	5	878	5.7	4.97 (1.80–13.7)	0.002	2.49 (0.91–6.77)	0.07

*Multivariable model was adjusted for sex, age, smoking status, index of multiple deprivation, rheumatic fever and atrial fibrillation.

†The Fine-Gray competing risks model was used to evaluate cause-specific mortality, with other causes of death modelled as a single competing outcome.

CVD, cardiovascular disease; MR, mortality rate per 1000 person-years; N, number at risk of death within category; n, number of deaths within category; PYs, person-years; VHD, valvular heart disease.

### All-cause mortality by VHD phenotype

Adjusted for other VHD phenotypes, mild tricuspid regurgitation (HR 1.34, 95% CI 1.06 to 1.69) and mild aortic regurgitation (HR 1.37, 95% CI 1.05 to 1.80) were associated with increased risk of all-cause mortality. However, neither remained significant once adjusted for age and other variables (tricuspid regurgitation HR 1.13, 95% CI 0.88 to 1.44; aortic regurgitation HR 1.09, 95% CI 0.083 to 1.44) (online supplemental table 6). Other individual VHD phenotypes were not associated with all-cause mortality.

### All-cause mortality in people with aortic sclerosis or MAC

In unadjusted KM survival analyses, the mean survival time was reduced in people with advanced aortic sclerosis or MAC compared with those in whom such disease was early or absent (p<0.0001) (figure 3, online supplemental figure 2).

**Figure 4:**
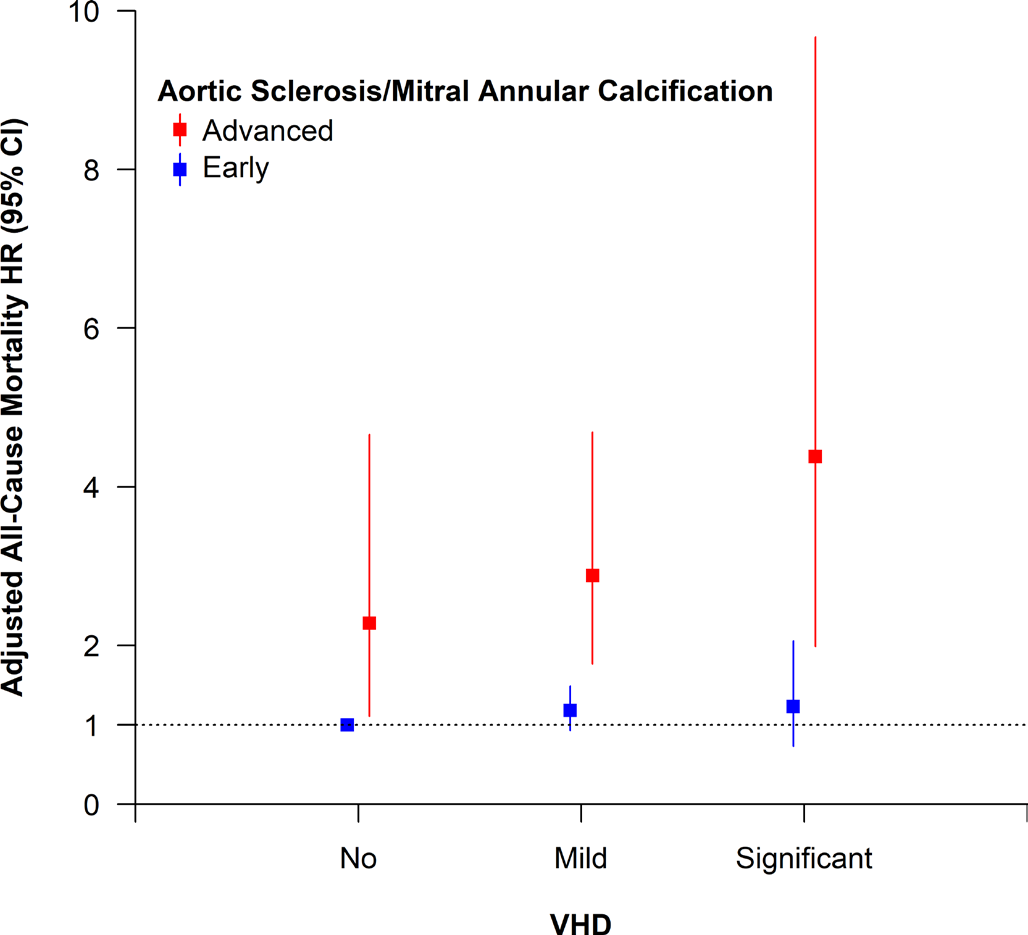
Association between VHD, calcific valve disease without functional effect (aortic sclerosis or MAC) and all-cause mortality (adjusted analysis). The HR and 95% CI shown are for combinations of no, mild or significant VHD with either early or advanced aortic sclerosis or MAC. Aortic sclerosis and MAC were collectively defined as manifestations of calcific valve disease without functional effect. People with aortic sclerosis could not have coexistent aortic stenosis (by definition), but could have other VHD. People with MAC could have associated mitral stenosis or regurgitation, or other VHD. VHD was categorised according to the affected valve, severity of valve lesion and associated clinical relevance (none/trivial, mild or clinically significant—the latter defined as moderate or severe stenosis or regurgitation). MAC, mitral annular calcification; VHD, valvular heart disease.

Even after adjustment for confounders, advanced aortic sclerosis (HR 2.05, 95% CI 1.28 to 3.30) and MAC (HR 2.51, 95% CI 1.41 to 4.49) remained associated with all-cause mortality, independent of other VHD phenotypes (figure 4, online supplemental table 6). All-cause mortality was even higher in people with advanced aortic sclerosis or MAC and coexistent clinically significant VHD affecting any valve (HR 4.38, 95% CI 1.99 to 9.67) compared with those with advanced aortic sclerosis or MAC and mild VHD (HR 2.88, 95% CI 1.77 to 4.69) or no VHD (HR 2.28, 95% CI 1.11 to 4.66) (table 3). In contrast, people with early or no aortic sclerosis or MAC were not at increased risk of death, even in the presence of clinically significant VHD.

**Table 3:** Association of VHD and either aortic sclerosis or mitral annular calcification with all-cause mortality

		Aortic sclerosis or mitral annular calcification							
		No/early				Advanced			
		N	n	HR (95% CI)	P value	N	n	HR (95% CI)	P value
Crude model									
VHD	No	1711	144	1.00 (reference)		40	8	2.85 (1.40 to 5.82)	0.004
	Mild	1512	166	1.46 (1.16 to 1.82)	0.001	66	19	4.46 (2.76 to 7.20)	<0.001
	Significant	166	17	1.90 (1.14 to 3.15)	0.013	16	7	13.7 (6.39 to 29.5)	<0.001
Multivariable model*									
VHD	No	1711	144	1.00 (reference)		40	8	2.28 (1.11 to 4.66)	0.024
	Mild	1512	166	1.18 (0.93 to 1.49)	0.17	66	19	2.88 (1.77 to 4.69)	<0.001
	Significant	166	17	1.23 (0.73 to 2.06)	0.44	16	7	4.38 (1.99 to 9.67)	<0.001

*Multivariable model was adjusted for sex, age, smoking status, index of multiple deprivation, rheumatic fever and atrial fibrillation.

N, number at risk of death within category; n, number of deaths within category; VHD, valvular heart disease.

## Discussion

### Summary of findings

We categorised aortic sclerosis and MAC as types of calcific valve disease without functional effect. These were common findings, particularly among people with clinically significant VHD (defined as moderate or severe stenosis or regurgitation) and the main driver of increased mortality. Advanced MAC and aortic sclerosis were associated with greater than double the risk of all-cause mortality, even in people without other VHD. Those with both clinically significant VHD and advanced aortic sclerosis or MAC had the worst outlook. These data suggest that atherosclerosis may be a key driver of the increased mortality risk in people with VHD.

Prevalence and severity of VHD were closely linked to increasing age. Mild VHD was very common but not associated with increased mortality. Clinically significant VHD was not associated with an increased risk of death in adjusted models, although relatively few cases were detected.

### Comparison with existing literature

There are limited data reporting survival rates according to the phenotype and severity of VHD, and existing studies have tended to focus on hospital populations with severe disease.^19^ A combined analysis of three North American population-based cohorts and an echocardiographic database found that patients with moderate and severe mitral or aortic VHD had 36%–75% increased risk of mortality compared with matched controls.^20^ A recent analysis of 25 827 people with aortic stenosis in the National Echocardiographic Database of Australia found that patients with any degree of aortic stenosis were at increased risk of death.^21^ In contrast, the OxVALVE study included people with all forms of VHD and found no definite association between clinically significant VHD and all-cause mortality after adjustment for confounders. This is likely to reflect the relatively few cases of clinically significant VHD detected, particularly those with aortic stenosis (the most malignant form of VHD).

There is also limited information reporting the survival of people with VHD detected at screening. The Echocardiographic Heart of England Screening (ECHOES) study recruited 6162 primary care patients who underwent echocardiographic screening for signs of heart failure or left ventricular systolic dysfunction.^22^ The 10-year survival of subjects with significant aortic or mitral valve disease was 25.8% compared with 76.1% among age-matched and sex-matched participants without VHD, heart failure or left ventricular systolic dysfunction.^22^ Our results provide contemporary mortality data categorised by valve abnormality and severity, and highlight the importance of combined forms of valve disease for prognostic estimates.

Both aortic sclerosis and MAC have previously been linked to increased mortality.^11 23 24^ In the Cardiovascular Health Study, people with aortic sclerosis had a 5-year mortality rate of 21.9% compared with 14.9% among people without aortic valve disease.^25^ A retrospective study including 3000 patients in California who underwent echocardiography between 1983 and 1998 reported that MAC was associated with a significantly increased risk of mortality in multivariate analysis (OR 2.50, 95% CI 1.91 to 3.45).^24^ Our results demonstrate the potential role of both aortic sclerosis and MAC as prognostic indicators in cardiovascular disease, alongside assessment of VHD.

### Strengths and limitations

To our knowledge, this is the first study to use linked national mortality registry data to report the causes of death in a large cohort of people with VHD. The OxVALVE-Survive study provides important information regarding the natural outcomes of VHD and the implications of screen-detected disease. Our findings can be generalised to other high-income countries where the prevalence of VHD and risk factors for death are likely to be comparable.

A single operator independently assessed each echocardiogram, thereby avoiding the risk of interobserver variability. Validation of the findings was not possible, and this may have introduced a degree of subjectivity into the assessment of VHD severity. However, this is consistent with clinical practice, and assessments were performed by an extremely experienced operator to minimise any discrepancy.

Cause of death was ascertained from linked ONS data that included information provided on the death certificate by the treating physician around the time of death. The recorded cause of death has been shown to differ from the cause of death at autopsy in up to a third of cases, and cardiovascular causes are often over-reported.^26^ However, all deaths in England must be registered by law and ONS remains one of the most globally reliable sources of mortality data. We adjusted for a focused set of covariates relevant to VHD. However, residual confounding resulting from unadjusted comorbidities remains a possibility since most deaths were non-cardiovascular in origin.

There were relatively few participants with clinically significant VHD, particularly aortic stenosis and bicuspid aortic valve disease, reflecting the fact that OxVALVE is a population screening study in an elderly population and that subjects with a previous diagnosis of VHD were excluded. Clinically significant VHD may have been associated with increased mortality had more patients with moderate or severe aortic stenosis been detected, given the established association with adverse clinical outcomes.^21^ The sample size was insufficient to allow analysis of outcomes according to every individual valve phenotype, particularly among participants with clinically significant VHD. Furthermore, data linkage was not possible for 498 participants in the original OxVALVE study and there were some differences in the baseline characteristics of this group compared with participants in the present analysis. However, the magnitude of these differences was small and unlikely to affect overall conclusions. Only 12 people underwent valve intervention, thereby precluding meaningful analysis of the impact of intervention on outcomes. Our results provide information on medium rather than long-term follow-up and further assessment of the OxVALVE cohort out to 20 years is planned.

OxVALVE collects data from a screened population, and while echocardiographic screening has been suggested as a means to detect VHD at an earlier stage this is not currently recommended practice.^5 12^ Our results support this policy, given we did not find an increased risk of death in people with screen-detected VHD. It is not possible to directly apply prognostic estimates from symptomatic cohorts to screen-detected patients (or vice versa), given the paucity of existing survival data and differences between the patient groups.

### Implications for policy and practice

Accurate mortality data linked to a well-phenotyped cohort support treatment planning for patients, clinicians and healthcare commissioners. The present survival analysis provides reassurance for those older people diagnosed with mild VHD (over 90% of those with any VHD) since it is unlikely to significantly alter their prognosis. Conversely, our demonstration of increased mortality in people with MAC and aortic sclerosis (both markers of atherosclerotic disease)^27^ indicates a need to establish whether intensive management of cardiovascular risk factors improves the prognosis of patients with calcific valve disease.

Utilisation of prognostic indicators to identify high-risk individuals may allow targeted treatment of modifiable risk factors. Recent studies have sought to identify new prognostic markers, such as the extent of aortic valve calcification or myocardial fibrosis, to refine prognostic estimates for people with aortic stenosis beyond standard clinical and echocardiographic parameters.^28^ However, aortic sclerosis and MAC are common and easily detectable, and represent potentially important prognostic indicators whose significance is often overlooked in clinical practice.

## Conclusion

Advanced aortic sclerosis or MAC was associated with a greater than twofold increase in risk of death, and this risk increased to fourfold in the presence of coexistent moderate and severe VHD. Mild VHD was common but not associated with an increased risk of all-cause mortality, thereby providing important reassurance to many patients.

**Key messages**

**What is already known on this subject?**

- Valvular heart disease (VHD) is common and prevalence is predicted to rise with population ageing.
- While some forms of moderate or severe disease have been linked to poor prognosis, VHD is often mild and of uncertain clinical importance.

**What might this study add?**

- In this prospective cohort study of 3511 screened participants, mortality was highest for people with both clinically significant VHD and advanced aortic sclerosis or mitral annular calcification (HR 4.38, 95% CI 1.99 to 9.67).
- Mild VHD was not associated with increased all-cause mortality (HR 1.20, 95% CI 0.96 to 1.51).

**How might this impact on clinical practice?**

- Older people with mild VHD can be reassured that this is unlikely to significantly alter their prognosis.
- Atherosclerosis appears to be an important driver of increased mortality in patients with clinically significant VHD and intensive risk factor modification may be warranted.

## Data Availability

Data are available upon reasonable request. Deidentified participant data from the OxVALVE population study were included in this analysis. Please contact the study team for queries about access to the data.

## References

[1] Coffey S , Cairns BJ , Iung B . The modern epidemiology of heart valve disease. Heart 2016;102:75–85. 10.1136/heartjnl-2014-307020 26541169

[2] Andell P , Li X , Martinsson A , et al . Epidemiology of valvular heart disease in a Swedish nationwide hospital-based register study. Heart 2017;103:1696–703. 10.1136/heartjnl-2016-310894 28432156 PMC5749343

[3] Olsen SJ , Fridlund B , Eide LS , et al . Changes in self-reported health and quality of life in octogenarian patients one month after transcatheter aortic valve implantation. Eur J Cardiovasc Nurs 2017;16:79–87. 10.1177/1474515116641297 27036955

[4] Brinkley DM , Gelfand EV . Valvular heart disease: classic teaching and emerging paradigms. Am J Med 2013;126:1035–42. 10.1016/j.amjmed.2013.05.022 24125637

[5] Baumgartner H , Falk V , Bax JJ , et al . 2017 ESC/EACTS guidelines for the management of valvular heart disease. Eur Heart J 2017;38:2739–91. 10.1093/eurheartj/ehx391 28886619

[6] Head SJ , Çelik M , Kappetein AP . Mechanical versus bioprosthetic aortic valve replacement. Eur Heart J 2017;38:2183–91. 10.1093/eurheartj/ehx141 28444168

[7] Rosenhek R , Klaar U , Schemper M , et al . Mild and moderate aortic stenosis. natural history and risk stratification by echocardiography. Eur Heart J 2004;25:199–205. 10.1016/j.ehj.2003.12.002 14972419

[8] Coffey S , d'Arcy JL , Loudon MA , et al . The OxVALVE population cohort study (OxVALVE-PCS)-population screening for undiagnosed valvular heart disease in the elderly: study design and objectives. Open Heart 2014;1:e000043. 10.1136/openhrt-2014-000043 25332795 PMC4195926

[9] d'Arcy JL , Coffey S , Loudon MA , et al . Large-scale community echocardiographic screening reveals a major burden of undiagnosed valvular heart disease in older people: the OxVALVE population cohort study. Eur Heart J 2016;37:3515–22. 10.1093/eurheartj/ehw229 27354049 PMC5216199

[10] Lancellotti P , Moura L , Pierard LA , et al . European association of echocardiography recommendations for the assessment of valvular regurgitation. Part 2: mitral and tricuspid regurgitation (native valve disease). Eur J Echocardiogr 2010;11:307–32. 10.1093/ejechocard/jeq031 20435783

[11] Yadgir S , Johnson CO , Aboyans V , et al . Global, regional, and national burden of calcific aortic valve and degenerative mitral valve diseases, 1990-2017. Circulation 2020;141:1670–80. 10.1161/CIRCULATIONAHA.119.043391 32223336

[12] Otto CM , Nishimura RA , Bonow RO , et al . 2020 ACC/AHA guideline for the management of patients with valvular heart disease: a report of the American College of Cardiology/American Heart Association Joint Committee on Clinical Practice Guidelines. Circulation 2021;143:e72–227. 10.1161/CIR.0000000000000923 33332150

[13] Gehlenborg N . UpSetR: a more scalable alternative to Venn and Euler diagrams for visualizing intersecting sets. R package version 1.4.0. 2019. Available: https://CRAN.R-project.org/package=UpSetR [Accessed 28 Oct 2019].

[14] Schisterman EF , Cole SR , Platt RW . Overadjustment bias and unnecessary adjustment in epidemiologic studies. Epidemiology 2009;20:488–95. 10.1097/EDE.0b013e3181a819a1 19525685 PMC2744485

[15] Shrier I , Platt RW . Reducing bias through directed acyclic graphs. BMC Med Res Methodol 2008;8:70. 10.1186/1471-2288-8-70 18973665 PMC2601045

[16] Therneau T . A package for survival analysis in S. version 2.38 2015. Available: https://CRAN.R-project.org/package=survivalaccessed28.10.19

[17] Kassambara A , Kosinski M . survminer: Drawing Survival Curves using 'ggplot2'. R package version 0.4.4, 2019. Available: https://CRAN.R-project.org/package=survminer [Accessed 29 Oct 2019].

[18] Gray B . cmprsk: Subdistribution analysis of competing risks. R package version 2.2-8, 2019. Available: https://CRAN.R-project.org/package=cmprsk [Accessed 29 Oct 2019].

[19] Iung B , Baron G , Butchart EG , et al . A prospective survey of patients with valvular heart disease in Europe: the Euro heart survey on valvular heart disease. Eur Heart J 2003;24:1231–43. 10.1016/S0195-668X(03)00201-X 12831818

[20] Nkomo VT , Gardin JM , Skelton TN , et al . Burden of valvular heart diseases: a population-based study. Lancet 2006;368:1005–11. 10.1016/S0140-6736(06)69208-8 16980116

[21] Strange G , Stewart S , Celermajer D , et al . Poor long-term survival in patients with moderate aortic stenosis. J Am Coll Cardiol 2019;74:1851–63. 10.1016/j.jacc.2019.08.004 31491546

[22] Taylor CJ , Roalfe AK , Iles R , et al . Ten-year prognosis of heart failure in the community: follow-up data from the echocardiographic heart of England screening (ECHOES) study. Eur J Heart Fail 2012;14:176–84. 10.1093/eurjhf/hfr170 22253455

[23] Di Minno MND , Di Minno A , Ambrosino P , et al . Cardiovascular morbidity and mortality in patients with aortic valve sclerosis: a systematic review and meta-analysis. Int J Cardiol 2018;260:138–44. 10.1016/j.ijcard.2018.01.054 29622430

[24] Ramaraj R , Manrique C , Hashemzadeh M , et al . Mitral annulus calcification is independently associated with all-cause mortality. Exp Clin Cardiol 2013;18:e5–7. 24294050 PMC3716491

[25] Otto CM , Lind BK , Kitzman DW , et al . Association of aortic-valve sclerosis with cardiovascular mortality and morbidity in the elderly. N Engl J Med 1999;341:142–7. 10.1056/NEJM199907153410302 10403851

[26] Pagidipati NJ , Gaziano TA . Estimating deaths from cardiovascular disease: a review of global methodologies of mortality measurement. Circulation 2013;127:749–56. 10.1161/CIRCULATIONAHA.112.128413 23401116 PMC3712514

[27] Massera D , Trivieri MG , Andrews JPM , et al . Disease activity in mitral annular calcification. Circ Cardiovasc Imaging 2019;12:e008513. 10.1161/CIRCIMAGING.118.008513 30712363 PMC6366554

[28] Clavel M-A , Pibarot P , Messika-Zeitoun D , et al . Impact of aortic valve calcification, as measured by MDCT, on survival in patients with aortic stenosis: results of an international registry study. J Am Coll Cardiol 2014;64:1202–13. 10.1016/j.jacc.2014.05.066 25236511 PMC4391203

