# Supplementary material for: Survival of people with valvular heart disease in a large, English community-based cohort study
Source: Heart. 2021 May 24;107(16):1336–43. doi: 10.1136/heartjnl-2020-318823 (PMC8327406; doi:10.1136/heartjnl-2020-318823)

Supplementary material: Survival of people with valvular heart disease in a large, English community-based cohort study

Supplementary Table 1 Classifications of mitral annular calcification and aortic sclerosis

**ECHO report**	**Classification**	**Mitral annular calcification**	**Aortic sclerosis (thickening and/or calcification)**
No	None	No involvement	0 - Normal (No involvement)
Mild	Early	Focal calcification with limited circumferential extent	1 - Mild (Minor involvement of one leaflet)
Moderate	Advanced	Marked calcification involving 1/3 to 1/2 of the mitral annular circumference;	2 - Moderate (Minor involvement of two leaflets or extensive involvement of one leaflet)
Severe		Marked calcification involving more than 1/2 of the mitral annular circumference, and/or intrusion into the LV outflow tract.	3 - Severe (Extensive involvement of two leaflets or involvement of all three leaflets)

Aortic sclerosis is defined as aortic valve cusp thickening without the development of a gradient across the aortic valve. The severity of aortic sclerosis on a scale of 0-3 (normal to severe) is quantified by echocardiography for echogenicity, thickening, or calcification of the valve leaflet as follows (Chandra et al., 2004).

Supplementary Table 2: Comparison of baseline clinical and echocardiographic characteristics of OxVALVE study participants according to the availability of long-term mortality data.

Characteristic	ONS linkage Yes		ONS linkage No		P-value
**N**	3511		498		
**Age, Mean (SD)**	72.64	(5.9)	75.11	(6.4)	**<0.0001**
**Men, n (%)**	1769	(50.4)	279	(56.0)	**0.009**
**Medical history**					
Angina, n (%)	261	(7.4)	48	(9.6)	0.07
Angiography, n (%)	282	(8.0)	54	(10.8)	**0.03**
Ankle Oedema, n (%)	493	(14.0)	91	(18.3)	**0.008**
Atrial Fibrillation, n (%)	224	(6.4)	32	(6.4)	0.91
Coronary Artery Bypass Graft, n (%)	57	(1.6)	12	(2.4)	0.19
Cerebrovascular Attack/Transient Ischaemic Attack, n (%)	199	(5.7)	45	(9.0)	**0.002**
Diabetes, n (%)	391	(11.1)	66	(13.3)	0.13
Hyperlipidaemia, n (%)	1305	(37.2)	206	(41.4)	0.13
Hypertension, n (%)	1556	(44.3)	248	(49.8)	**0.010**
Myocardial Infarction, n (%)	157	(4.5)	33	(6.6)	**0.03**
NYHA Class, n (%)					**0.003**
I	2811	(80.1)	372	(74.7)	
II	629	(17.9)	97	(19.5)	
III-IV	71	(2.0)	22	(4.4)	
Percutaneous Coronary Intervention, n (%)	135	(3.9)	18	(3.6)	0.85
Rheumatic Fever, n (%)	73	(2.1)	11	(2.2)	0.82
**Smoking Status, n (%)**					**0.02**
Non-Smoker	1883	(53.6)	230	(46.2)	
Ex-Smoker	1396	(39.8)	226	(45.4)	
Smoker	230	(6.6)	35	(7.0)	
**Index of multiple deprivation (quintile), n (%)**					**0.003**
1 (least deprived)	1029	(29.3)	111	(22.3)	
2	1228	(35.0)	247	(49.6)	
3	749	(21.3)	75	(15.1)	
4	364	(10.4)	54	(10.8)	
5 (most deprived)	127	(3.6)	4	(0.8)	
**Examination**					
Height, Mean (SD)	1.67	(0.10)	1.66	(0.09)	**0.02**
Weight, Mean (SD)	77.8	(15.7)	74.57	(15.0)	**<0.0001**
Body Mass Index, Mean (SD)	27.7	(4.8)	26.9	(4.7)	**0.0004**
Systolic Blood Pressure, Mean (SD)	143.7	(20.2)	139.8	(20.2)	**<0.0001**
Diastolic Blood Pressure, Mean (SD)	80.4	(11.4)	78.7	(11.9)	**0.004**
Heart rate, Mean (SD)	72.8	(12.1)	73.1	(11.9)	0.62
**Echocardiography**					
Mitral Regurgitation, n (%)	1009	(28.7)	111	(23.5)	**0.012**
Mitral Stenosis, n (%)	5	(0.14)	5	(2.2)	**0.0004**
Aortic Regurgitation, n (%)	579	(16.5)	87	(18.7)	0.40
Aortic Stenosis, n (%)	40	(1.1)	11	(3.4)	0.11
Aortic Sclerosis, n (%)	1653	(47.0)	195	(40.4)	**0.0002**
Tricuspid Regurgitation, n (%)	1074	(30.6)	144	(30.1)	0.24
Pulmonary Regurgitation, n (%)	18	(0.51)	3	(1.8)	0.78
Bicuspid Aortic, n (%)	8	(0.23)	2	(1.6)	0.46
Mitral Prolapse, n (%)	59	(1.7)	4	(2.0)	0.32
Mitral Annular Calcification, n (%)	463	(13.2)	66	(14.5)	0.06

Supplementary Table 3 Valvular heart disease phenotypes ranked according to frequency (moderate and severe disease are grouped together as advanced (aortic sclerosis or mitral annular calcification) or significant disease (Valvular Heart Disease).

Characteristic	n	(%)
**Aortic sclerosis, n (%)**		
No	1858	(52.92)
Early	1574	(44.83)
Advanced	79	(2.25)
**Tricuspid regurgitation, n (%)**		
No	2437	(69.41)
Mild	1009	(28.74)
Significant	65	(1.85)
**Mitral regurgitation, n (%)**		
No	2502	(71.26)
Mild	941	(26.80)
Significant	68	(1.94)
**Aortic regurgitation, n (%)**		
No	2932	(83.51)
Mild	524	(14.92)
Significant	55	(1.57)
**Mitral annular calcification, n (%)**		
No	3048	(86.81)
Early	417	(11.88)
Advanced	46	(1.31)
**Mitral valve prolapse, n (%)**		
None/trivial	3452	(98.32)
Mild	55	(1.57)
Significant	4	(0.11)
**Aortic stenosis, n (%)**		
No	3471	(98.86)
Mild	21	(0.60)
Significant	19	(0.54)
**Pulmonary regurgitation, n (%)**		
No /Mild	3493	(99.49)
Significant	18	(0.51)
**Bicuspid aortic valve, n (%)**		
None/trivial/Mild	3503	(99.77)
Significant	8	(0.23)
**Mitral stenosis, n (%)**		
No	3506	(99.86)
Mild	4	(0.11)
Significant	1	(0.03)

Supplementary Table 4 Valvular heart disease phenotypes ranked according to frequency and severity of disease.

	**None/trivial**		**Mild**		**Moderate**		**Moderate-to-Severe**		**Severe**	
**VHD Subtype**	**N**	**%**	**N**	**%**	**N**	**%**	**N**	**%**	**N**	**%**
Aortic sclerosis	1858	52.9	1574	45.0	73	2.08	0	0	6	0.17
Tricuspid regurgitation	2437	69.4	1009	29.0	49	1.40	5	0.14	11	0.31
Mitral regurgitation	2502	71.3	941	27.0	59	1.68	7	0.20	2	0.06
Aortic regurgitation	2932	83.5	524	15.0	52	1.48	3	0.09	0	0
Mitral annular calcification	3048	86.8	417	12.0	45	1.28	0	0	1	0.03
Mitral prolapse	3452	98.3	55	1.60	4	0.11	0	0	0	0
Aortic stenosis	3471	98.9	21	0.60	14	0.40	1	0.03	4	0.11
Pulmonary regurgitation	3493	99.5	0	0	18	0.51	0	0	0	0
Bicuspid aortic valve	3503	99.8	0	0	8	0.23	0	0	0	0
Mitral stenosis	3506	99.9	4	0.10	0	0	1	0.03	0	0

Supplementary Table 5 Number and causes of death for OxVALVE study participants with significant, mild and no valvular heart disease.

	VHD									
**Cause of death**	**Significant**	**%**	**Mild**	**%**	**None**	**%**	**Any VHD**	**%**	**Overall**	**%**
Any	24	100	185	100	152	100	361	100	361	100
Cancer	3	12.5	76	41.8	79	52.0	79	21.9	158	43.8
Cardiovascular disease										
Primary cause of death	8	33.3	44	24.2	34	22.4	52	14.4	86	23.8
Any cause of death	12	50.0	94	51.7	65	42.8	106	29.4	171	47.4
Respiratory disease	5	20.8	19	10.4	14	9.21	24	6.6	38	10.5
Other causes of death	8	33.3	43	23.6	25	16.5	51	14.1	76	21.1

* Causes of death affecting <10 individuals in either of the groups being compared were grouped together.

There were three individuals for whom we could not obtain the cause of death.

Supplementary Table 6 Association of individual valvular heart disease phenotypes with all-cause mortality

**Valve disease subtype**	**Category**	**N**	**n**	**PYs**	**MR**	**Model 1[a]**	**Model 2[b]**
Valvular heart disease sub-types							
**Tricuspid regurgitation**	No	2437	229	15,603	14.7	1.00 Reference	1.00 Reference
	Mild	1009	125	6,169	20.3	1.34 (1.06 to 1.69)	1.13 (0.88 to 1.44)
	Significant	65	7	298	23.5	1.15 (0.48 to 2.73)	0.73 (0.30 to 1.80)
**Mitral regurgitation**	No	2502	251	16,241	15.5	1.00 Reference	1.00 Reference
	Mild	941	99	5,524	17.9	1.01 (0.79 to 1.3)	0.95 (0.74 to 1.23)
	Significant	68	11	305	36.1	1.93 (0.96 to 3.86)	1.48 (0.73 to 3.03)
**Aortic regurgitation**	No	2932	282	18,608	15.2	1.00 Reference	1.00 Reference
	Mild	524	74	3,216	23.0	1.37 (1.05 to 1.80)	1.09 (0.83 to 1.44)
	Significant	55	5	245	20.4	1.15 (0.46 to 2.89)	0.87 (0.34 to 2.25)
Other valve abnormalities							
**Mitral annular calcification**	No	3048	285	19,256	14.8	1.00 Reference	1.00 Reference
	Early	417	63	2,571	24.5	1.55 (1.16 to 2.05)	1.34 (1.01 to 1.78)
	Advanced	46	13	243	53.5	3.36 (1.92 to 5.89)	2.51 (1.41 to 4.49)
**Aortic sclerosis**	No	1858	1982	12,689	15.1	1.00 Reference	1.00 Reference
	Early	1574	148	8,953	16.5	0.98 (0.78 to 1.24)	0.83 (0.66 to 1.06)
	Advanced	79	21	427	49.1	2.79 (1.74 to 4.46)	2.05 (1.28 to 3.30)

Abbreviations: **N**: number at risk of death within category; **n**: number of deaths within category; **PYs**: person-years; **MR**: Mortality rate per 1,000 person-years.

[a] Model 1 was adjusted for all other VHD subtypes.

[b] Model 2 was adjusted for all other VHD subtypes and sex, age, smoking status, index of multiple deprivation, blood pressure (systolic and diastolic), diabetes, and rheumatic fever.

Supplementary Figure 1 Simplified directed acyclic graph

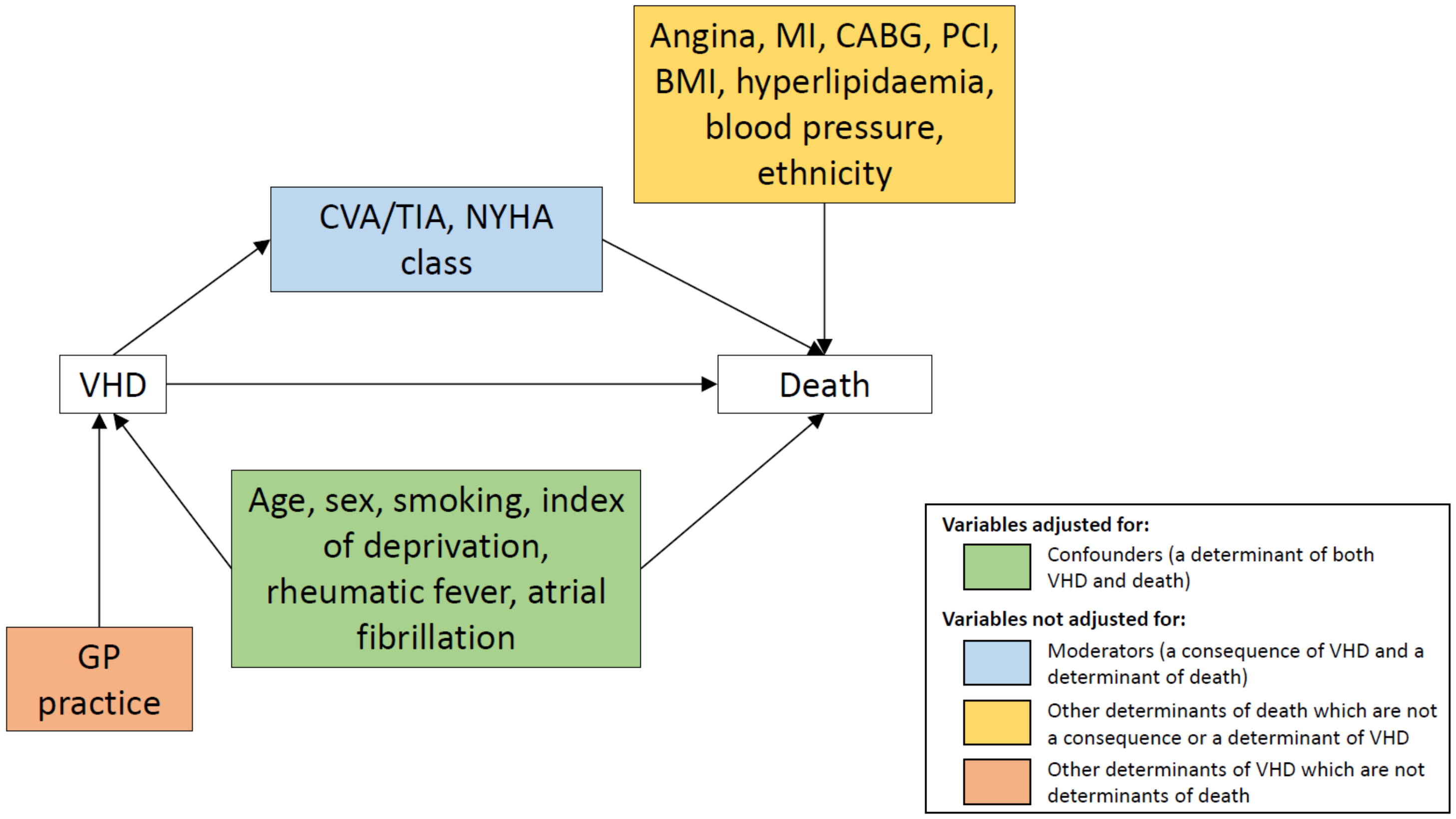

Supplementary Figure 2 Kaplan-Meier curves demonstrating the unadjusted rates of all-cause mortality for people with aortic sclerosis (AoScl) or mitral annular calcification (MAC).

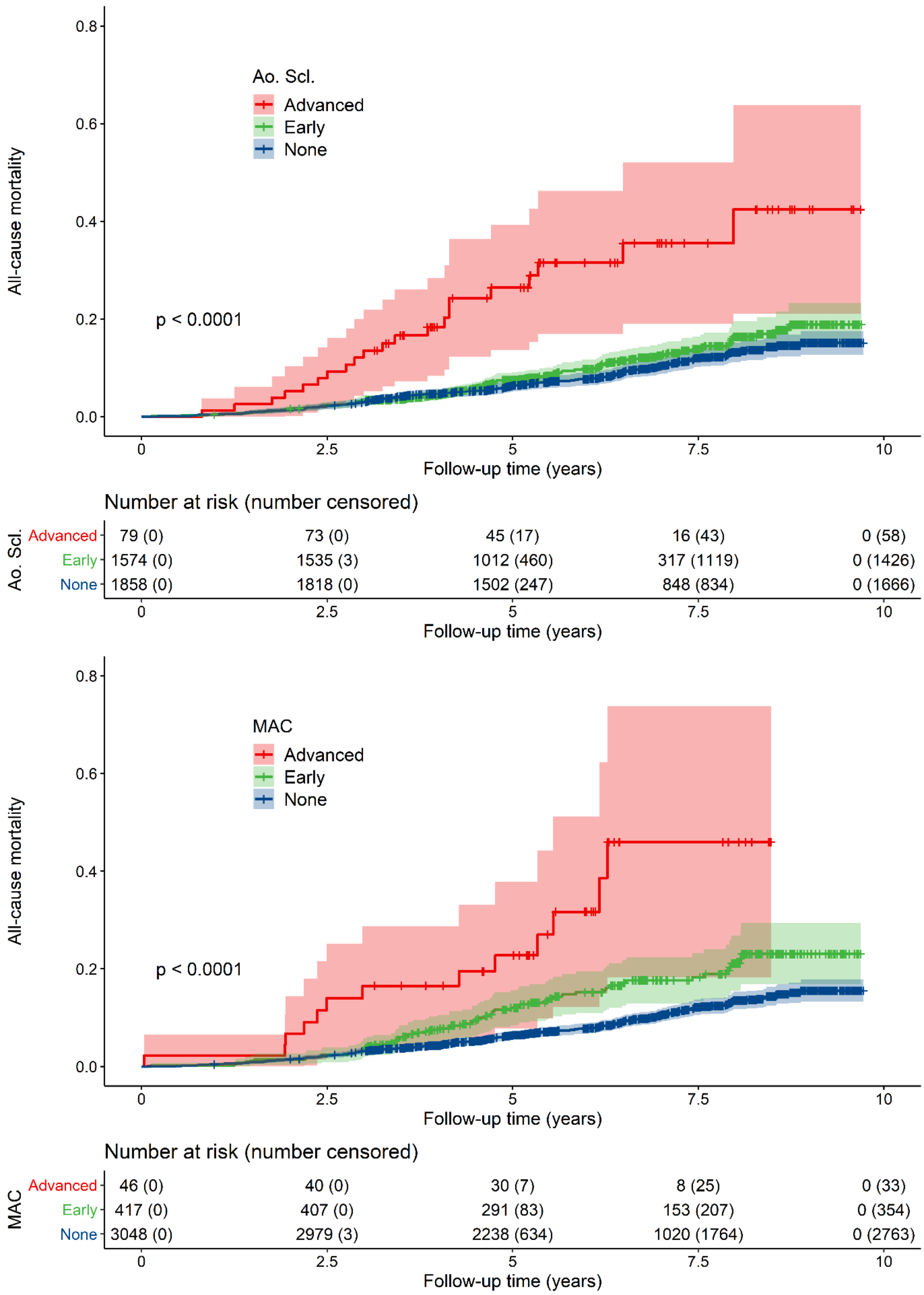

Supplement: Supplementary data [file heartjnl-2020-318823supp001.pdf]
